# Porphyrin-Based Metal-Organic Framework Materials: Design, Construction, and Application in the Field of Photocatalysis

**DOI:** 10.3390/molecules29020467

**Published:** 2024-01-17

**Authors:** Chuanyin Tang, Xiaoyu Li, Yingxu Hu, Xin Du, Shuo Wang, Bo Chen, Shengjie Wang

**Affiliations:** College of Chemistry and Chemical Engineering, China University of Petroleum, Qingdao 266580, China; s22030166@s.upc.edu.cn (C.T.); s21030109@s.upc.edu.cn (X.L.); s23030143@s.upc.edu.cn (Y.H.); z21030160@s.upc.edu.cn (X.D.); uzicorn@163.com (S.W.); 2203050217@s.upc.edu.cn (B.C.)

**Keywords:** metal-organic frameworks, porphyrin, photoelectric transmission, relationship between the structure and property, photocatalytic applications

## Abstract

Metal-organic frameworks (MOFs) are a novel category of porous crystalline materials with an exceptionally high surface area and adjustable pore structure. They possess a designable composition and can be easily functionalized with different units. Porphyrins with conjugated tetrapyrrole macrocyclic structures can absorb light from ultraviolet to visible light regions, and their structures and properties can be facilely regulated by altering their peripheral groups or central metal ions. Porphyrin-based MOFs constructed from porphyrin ligands and metal nodes combine the unique features of porphyrins and MOFs as well as overcoming their respective limitations. This paper reviewed the design and construction, light absorption and charge transfer pathways, and strategy for improving the photocatalytic performance of porphyrin-based MOFs, and highlighted the recent progress in the field of CO_2_ reduction, hydrogen evolution, organic synthesis, organic pollutant removal, and nitrogen fixation. The intrinsic relationships between the structure and the property of porphyrin-based MOFs received special attention, especially the relationships between the arrangements of porphyrin ligands and metal nods and the charge transfer mechanism. We attempted to provide more valuable information for the design and construction of advanced photocatalysts in the future. Finally, the challenges and future perspectives of the porphyrin-based MOFs are also discussed.

## 1. Introduction

The utilization of fossil energy brings great benefits to human beings. However, over reliance on traditional fossil energy such as coal and petroleum results in serious problems such as energy shortages and environment pollution [1,2]. People search for alternative energy resources to reduce their reliance on traditional fossil fuels [3,4,5]. As is known, solar energy is an important alternative energy source. About 120,000 terawatts (TW) of solar energy reaches the earth’s surface every year, far exceeding the global total energy demand [6]. Moreover, solar energy has emerged as a crucial sustainable energy resource due to its abundance, cleanliness, affordability, and inexhaustibility. However, efficient capturing and utilization of solar energy is still challenging because of its lower energy density and its being affected by the weather, season, and geographical position. Therefore, solar energy is converted to thermal energy, electrical energy, and chemical energy to solve the problem of direct utilization.

Photocatalytic technology is one of the most promising methods to solve both environmental and energy problems due to its simple reaction conditions, low cost, lack of secondary pollution, and environmental protection [7], and it exhibits extensive applications in hydrogen evolution, nitrogen fixation, CO_2_ reduction, as well as the elimination of organic and inorganic pollutants. The design and development of efficient photocatalysts attract great attention since photocatalysts play a crucial role in photocatalytic reactions [8]. So far, metal oxides [9,10], metal phosphides [11,12], metal sulfides [13,14,15], and carbon-based composites [16,17,18,19] have been developed and used in energy conversion systems. However, these traditional photocatalysts suffered from different challenges such as low electronic conductivity, low apparent quantum efficiency, rapid recombination of electron-hole pairs, poor chemical stability and cycle stability, and so on [20]. Therefore, the construction of photocatalysts with high performance and physical/chemical stability holds great promise in addressing the current energy and environmental issues.

Metal-organic frameworks (MOFs) are crystalline nanoporous hybrid materials composed of metal nodes connected by organic ligands [21]. They can be modularly assembled with suitable inorganic and organic building units, allowing for the design of functional frameworks with specific topologies, adjustable porosity, and desirable properties [22]. Different from other porous compounds such as zeolites, MOFs are easily modifiable. The products can be obtained under relatively simple conditions and thus obtain good specificity and selectivity. The rich coordination chemistry of MOFs enables the adjustment of their photoelectric properties by changing their composition [23]. In this context, MOFs can be widely used in various fields including gas storage and separation [24,25], photocatalysis [22,26,27], sensors [28,29], pollutant degradation [30,31], ion exchange [32], and so on.

Porphyrin molecules have unique photophysical and electrochemical properties, as well as remarkable visible light absorption and energy transfer characteristics [33,34,35,36,37]. Using porphyrin as an organic ligand of MOFs, porphyrin molecules can be uniformly dispersed in precisely adjustable MOFs. The incorporation of porphyrin endows the MOFs with unique optical properties and catalytic activity and at the same time, overcomes some inherent drawbacks of porphyrin molecules, such as the tendency towards self-aggregation and light instability [32].

Moreover, MOFs are believed to be an ideal platform for solar light absorption and energy transfer in artificial photosystems owing to their repeated and order arrangement [38]. The introduction of porphyrin units into MOFs can broaden their absorption spectrum to the visible light range. Therefore, porphyrin-based MOFs obtained by integrating porphyrins with metal nodes become multifunctional carriers with excellent photocatalytic performance and desired functionality [39,40]. Thus, porphyrin-based MOFs have received significant attention due to their tremendous potential in various fields, such as photocatalysis [41,42,43], biomedicine [44,45,46,47], electrochemistry [48,49,50,51], and molecular adsorption [52]. Especially in the design of new photocatalytic materials, the photocatalytic activity of porphyrin-based MOFs can be regulated by the design of porphyrin molecule structure, and can also be optimized by the co-structure design of mixed ligands in MOF and the co-assembly doping design with other materials. In this paper, we mainly review the design and construction of porphyrin-based MOFs and their applications in the fields of photocatalytic generation of hydrogen, CO_2_ reduction, organic synthesis, and degradation of pollutants. We also discuss the research direction in the future and potential applications of porphyrin-based MOFs.

## 2. Photocatalytic Mechanism

### 2.1. Light Absorption

**Figure 1:**
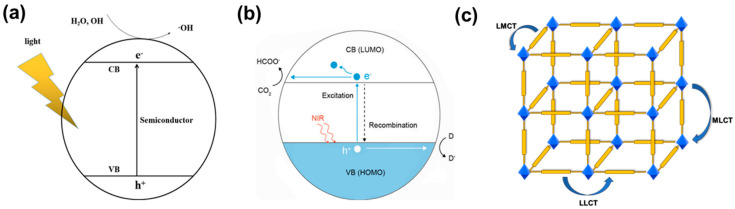
(**a**) Photocatalytic mechanism of the semiconductor [56]; (**b**) mechanism of MOF photocatalytic reduction of CO_2_ [57]; (**c**) schematic diagram of charge transfer pathways in MOFs, such as LMCT, LLCT, and MLCT.

The light absorption of semiconductors is determined by their band gap. According to the band theory, the energy gap of MOFs depends on the distance between the highest occupied molecular orbital (HOMO) and the lowest unoccupied molecular orbital (LUMO) energy levels, which correspond to VB and CB of inorganic semiconductors [20,53,54], respectively (Figure 1a,b). Photocatalysts absorb light energy and facilitate the transition of electrons from HOMO to LUMO. Charge transfer from ligand to metal cluster or ligand (n→π*, π→π*) constitutes the majority of the charge transition in MOFs under irradiation (Figure 1c) [55].

In addition, light-responsive characteristics can be easily regulated by a reasonable selection of ligands and metal ions. As one of the most commonly used photosensitive organic ligands that have large π-bonding [58], porphyrin exhibits great potential in improving light response owing to its high molar extinction coefficient. The molar extinction coefficient is a measure of a substance’s ability to absorb light, and it can be used to describe the strength of a material’s absorption of light at different wavelengths. The porphyrin molecule itself contains a large number of conjugated double bonds and aromatic ring structures, which endows it with a higher molar extinction coefficient than most organic ligands used in MOFs. Therefore, porphyrin molecules exhibit excellent light-harvesting capabilities and great potential in light-responsive MOFs. Typically, porphyrins absorb light at 400–450 nm (Soret band) and 500–650 nm (Q bands). Interestingly, the incorporation of porphyrin within MOFs results in a wider absorption region in visible light region [59]. This endows porphyrin-based MOFs with improved light absorption capacity.

### 2.2. Charge Transfer Pathway 

The combination of organic ligands and metal clusters in MOFs endows them with semiconductor behavior and a similar principle of photocatalysis [60,61]. Metal clusters and photoactive ligands absorb light energy and result in photo-induced charge separation, that is, electrons with reducing ability e^−^ and holes with oxidizing ability h^+^. Then the photo-generated charges migrate to the surface of the photocatalyst to participate in the redox reaction. That is to say, metal clusters accept electrons generated by organic ligands for subsequent redox reactions [62]. Transferring e^−^ from the highest occupied molecular orbital (HOMO) of organic ligands to the lowest unoccupied molecular orbital (LUMO) of metal clusters is labeled as the ligand-to-metal charge transfer (LMCT) pathway in MOFs [63]. Studies have shown that the LMCT pathway plays an important role in enhancing the charge separation and photocurrent density in MOFs, and therefore is beneficial to enhancing their photocatalytic ability [64]. There are two pathways including the ligand-to-ligand charge transfer (LLCT) and metal-to-ligand charge transfer (MLCT) in addition to the LMCT pathway (Figure 1c) [65,66]. The photo-generated electrons and holes through the above-mentioned pathways will proceed to reduce and oxidize the substances adsorbed on the surface of the MOFs, and then produce different products in the processes of photocatalytic hydrogen evolution, CO_2_ reduction, organic synthesis, pollutant degradation, and nitrogen fixation [67,68].

## 3. Design and Construction of Porphyrin-Based MOFs 

### 3.1. Design of Porphyrin-Based MOFs 

Some drawbacks of MOFs including rapid recombination and deactivation of photo-generated charges as well as poor cycling stability greatly impede their large-scale application in photocatalysis; although, MOFs and their composite materials possess advantages of tunable pores, high porosity, and a large surface area. These limitations hinder the efficient utilization and long-term stability of MOFs in photocatalytic processes. Porphyrins and their derivatives have excellent light absorption capacity and are easily coordinated with metal ions, which facilitates addressing these problems of MOFs. Therefore, the incorporation of porphyrins into MOF materials endows them with the advantages of porphyrin molecules and MOF materials. In addition, MOFs with various topologies and functions can be obtained by the design of the connectivity and geometry of the porphyrin linker. 

The chemical properties and coordination abilities can also be regulated by introducing different functional groups onto porphyrin molecules. For example, the electron affinity and electron conductivity of porphyrins can be changed by the introduction of various substituents or modified peripheral groups [69,70,71]. This modification allows for the attainment of appropriate orbital energy levels and band gaps, and results in the construction of porphyrin-based MOFs with exceptional light-harvesting capabilities and efficient charge separation and transfer efficiencies. 5,10,15,20-tetrakis(4-carboxyphenyl) porphyrin (TCPP) with the capacity to coordinate with Zr, Cu, Al, Cr, La, and other metals, is the most commonly used organic linker in porphyrin-based MOFs. Moreover, the incorporation of metal ions helps to address the issues of low stability, poor dispersion, and easy aggregation of MOFs, and results in enhanced photocatalytic performance.

### 3.2. Construction of Porphyrin-Based MOFs 

**Figure 2:**
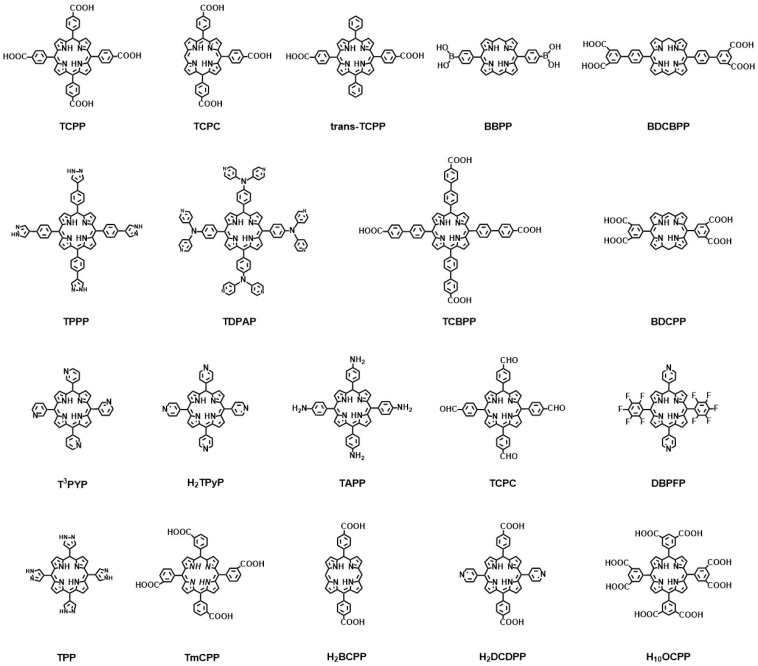
Representative chemical structures of porphyrin ligands in porphyrin-based MOFs.

In porphyrin-based MOFs, porphyrin or metal porphyrin molecules act as organic ligands to coordinate with metal ions or metal clusters. The porphyrin linkers in porphyrin-based MOFs mainly include carboxylic acids, pyridines, and polyazole groups [72,73,74]. The following are most of the porphyrin ligands that are commonly used in porphyrin-based MOFs, shown in Figure 2 [75,76,77,78,79,80,81,82]. 

#### 3.2.1. Porphyrin-Based MOFs with Carboxylic Acid Linkers

The porphyrin ligand with a carboxylic acid linker is the most widely used in the construction of porphyrin-based MOFs. It is connected to the metal center through a flexible coordination mode, resulting in a controllable and adjustable ligand structure. Such porphyrin-based MOFs usually exhibit high stability and controllability in structures and are suitable for application in gas adsorption, catalysis, and photocatalysis.

**Figure 3:**
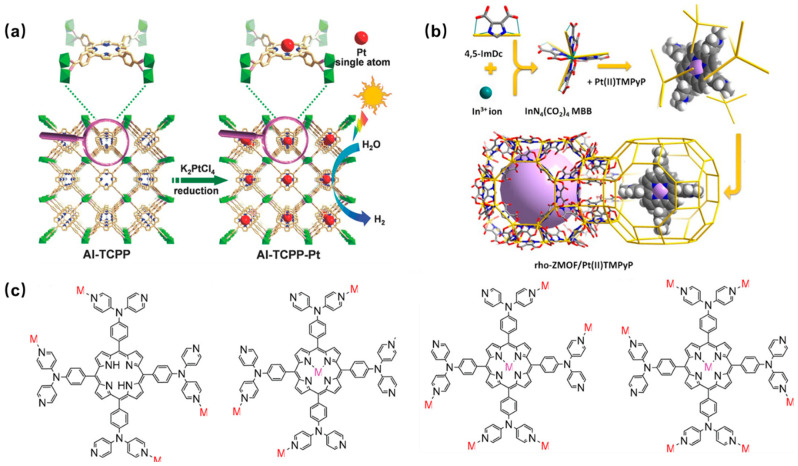
(**a**) Schematic diagram of the preparation of Al-TCPP-Pt photocatalyst [83]; (**b**) synthesis method of Pt(II)TMPyP/rho-ZMOF composites [88]; (**c**) four coordination modes of porphyrin TDPAP [89].

An atomically Pt-dispersed aluminum-porphyrin-based MOF (Al-TCPP-Pt) was synthesized using tetracarboxylic porphyrin (H_2_TCPP) as the organic ligand to interconnect Al(OH)O_4_ chains by a hydrothermal reaction and a subsequent Pt(II) metalation [83]. The hybrid Al-TCPP-Pt MOFs showed significantly enhanced photocatalytic activity, utilizing the highly efficient electron transfer channel provided by platinum (Pt) atoms (Figure 3a). Similarly, alkaline earth-porphyrin-based MOFs [Sr_4_(TCPP)_2_(DMF)_8_]_n_ and [Ba_4_(TCPP)_2_(DMF)_8_]_n_ were synthesized using H_4_TCPP ligand and Sr^2+^/Ba^2+^ ions by hydrothermal reaction [84]. The PMOFs exhibited excellent fluorescence properties. The size and morphology of the MOFs were altered by replacing the TCPP ligand with 5,15-bis(4-carboxyphenyl) porphyrin (H_2_DCPP); thus, a series of four porphyrin alkaline earth-based MOFs were obtained. They exhibited excellent adsorption capabilities for methylene blue [85].

The nucleation and growth rate of nano-MOFs can be regulated by adjusting the ratio of metal ions to organic ligands, solvent type, reaction temperature, pH value, reaction time, and other conditions. A series of MOFs based on carboxylic acid zirconium-porphyrin, namely PCN-222, PCN-223, and PCN-224, were synthesized by changing the experimental conditions. They were used as CO_2_-reducing photocatalysts under visible light irradiation. The results showed that PCN-224 with the lowest linker connectivity exhibited a higher catalytic performance compared to that of PCN-222 and PCN-223 which had higher node connectivity [86].

#### 3.2.2. Porphyrin-Based MOFs with Nitrogen-Containing Heterocyclic Linkers

Nitrogen-containing heterocyclic ligands include imidazole, polyazole, pyridine, pyrazole ligands, and so on. The coordination strength between nitrogen atoms and metal ions is greater than that between oxygen atoms and metal ions. Therefore, MOFs composed of nitrogen-containing heterocyclic ligands exhibit comparable chemical and thermal stability to carboxylic acid-based MOFs. At the same time, nitrogen-containing heterocyclic MOFs may exhibit better stability than carboxylic MOFs under extreme pH conditions through appropriate metal-ligand matching.

In 1994, Robson and coauthors [87] first reported a coordinated framework material containing Cu(II) and neutral pyridyl-substituted porphyrin units. Since then, an increasing number of porphyrin-based MOFs have been reported. Pyridine-containing porphyrin-based MOFP was prepared by encapsulating platinum metalized porphyrin (Pt(II)TMPyP) in a rho zeolite-like metal-organic framework (rho-ZMOF) via a post-modification method (Figure 3b). They exhibited excellent sensitivity and selectivity for various anions in both aqueous and methanol media, leading to a significant enhancement in the detection limit of anions [88]. In addition, a novel pyridine-containing porphyrin ligand 5,10,15,20-tetrakis (4,4-dipyridylaminophenylene) porphyrin (TDPAP) was designed and synthesized, and introduced four peripheral 4,4′-bipyridylamine moieties in the porphyrin platform (Figure 3c) [89]. The novel-designed porphyrin molecule contains eight peripheral pyridines, so it can coordinate with up to nine metal centers. Different MOFs were formed using a solvothermal method with TDPAP and various metal ions such as Mn^2+^, Cu^2+^, Zn^2+^, and Cd^2+^ metal ions. These materials show excellent fluorescence properties except for the MOFs prepared from Mn^2+^.

Pyrazole carboxylate is another nitrogen-containing heterocyclic group similar to imidazole. In the pyrazole carboxylate connectivity, the coordination between the linker and the metal cluster occurs through the sp-hybridized nitrogen atom and the hydrogen bonding between the adjacent NH group and the carbonyl moiety of the metal cluster. For example, PCN-601 and PCN-602, pyrazole carboxylate-based porphyrin MOFs, exhibit excellent stability even in highly alkaline conditions. Their porosity and crystallinity are kept intact even after treatment with a saturated NaOH solution at 100 °C [90,91].

## 4. Improvement in Photocatalytic Performance of Porphyrin-Based MOFs

### 4.1. Modification of Porphyrin Ligands

As one of the important components of MOFs, organic ligands play a crucial role in determining their topological structure, pore size, shape, and surface properties. They serve as the main supporting unit of the framework and their size and shape affect the aforementioned characteristics [92,93]. In addition, organic ligands in MOFs act as ‘photosensitive antennas’, determine the light-capturing ability, and are responsible for the charge transfer processes of LMCT or MLCT pathways [94,95]. Therefore, the rational selection and modification of organic ligands can remarkably improve the photocatalytic ability of MOFs. As the most commonly used photosensitive ligand with a large π structure, porphyrin has become an important target for ligand modification and hybridization strategies. It is well known that incorporating ligands with a large π structure or photosensitive units in MOFs can enhance their light response and charge transfer efficiency. Selecting porphyrin molecules as organic ligands for MOFs and modifying them is of great significance in improving the photocatalytic ability of MOFs.

#### 4.1.1. The Introduction of Metal Coordination Center

The modification of porphyrin ligands mainly refers to the insertion of metal ions into the porphyrin core to form metalloporphyrin complexes [96]. After metalation, the coordination bonds are formed between the metal ion and the porphyrin ring which enhances the symmetry of the porphyrin molecule, resulting in stronger absorption in the visible and near-infrared regions and suppressed recombination of photo-generated electron-hole pairs [97,98,99,100,101,102,103]. The optical properties and catalytic activity of metalloporphyrin-based MOFs can be regulated by selecting different metal ions and controlling the structure of the porphyrin ligands.

**Figure 4:**
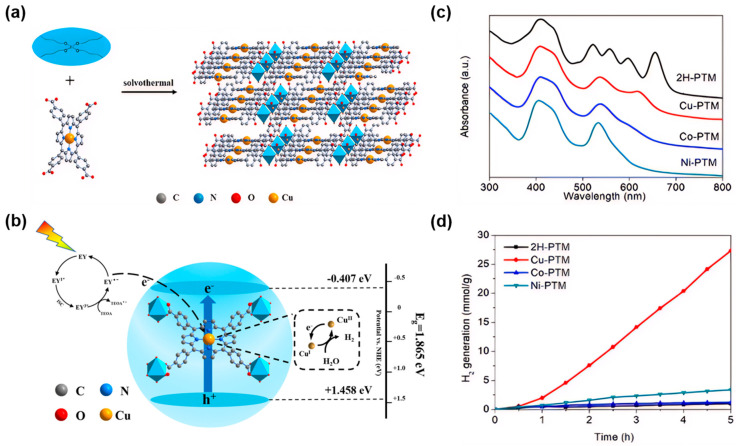
(**a**) Synthesis of Cu-PTM [104]; (**b**) photocatalytic mechanism of Cu-PTM for hydrogen evolution under visible light [104]; (**c**) UV-visible spectra of 2H-PTM, Cu-PTM, Co-PTM, and Ni-PTM [104]; (**d**) photocatalytic hydrogen evolution diagrams in the presence of 2H-PTM, Cu-PTM, Co-PTM, and Ni-PTM photocatalysts [104].

The stability and biocompatibility of the materials can be enhanced to meet the requirements for different applications. For example, a new ligand-to-linker metal charge-transfer (LLMCT) pathway was found by anchoring non-precious metal Cu in the center of the porphyrin in Ti-porphyrin-based MOFs (Figure 4a,c). The photo-generated electrons initially originated from the excited eosin Y (EY*) can transfer from the lowest unoccupied molecular orbital (LUMO) level of the porphyrin to the transient Cu^I^/Cu^II^ center via rapid delocalization through the conjugated 18-π electron system of the porphyrin. This can reduce the width of the bandgap and at the same time, improve the charge separation and transfer rate, thereby enhancing the charge transfer efficiency (Figure 4b). Experimental results show that the addition of metal ions in MOFs can achieve a hydrogen evolution rate of 5465.689 μmol g^−1^ h^−1^, approximately 27.71 times that of the pristine MOFs (Figure 4d), indicating the Cu in porphyrin core plays a key role in improving the photocatalytic performance [104].

#### 4.1.2. Incorporation of Functional Groups

**Figure 5:**
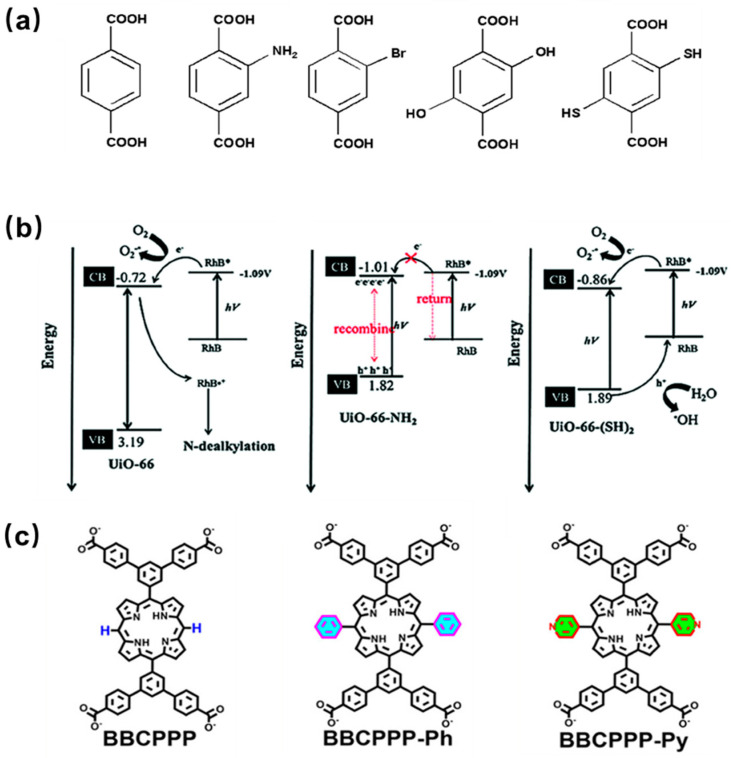
(**a**) Molecule structure of functionalized terephthalic acid linker, H_2_BDC-X [106]; (**b**) band gap diagram, energy transfer between UiO-66 and UiO-66-NH_2_, and the degradation mechanism of Rhodamine B over UiO-66-(SH)_2_ [106]; (**c**) the chemical structures of BBCPPP, BBCPPP-Ph, and BBCPPP-Py [108].

The structures and properties of MOFs depend on the organic ligands and metal clusters and can be regulated by the incorporation of active ingredients. In addition, by functionalizing organic linkers (ligands) with other groups (such as amino), the light capture properties of MOFs can be regulated [105]. Therefore, the incorporation of functional groups on the ligand can optimize the structure and properties of the materials, such as increasing the porosity and surface area, decreasing the band gap, and thus improving the catalytic performance of MOFs. A series of isomorphic UiO-66-X (X = H, NH_2_, Br, (OH)_2_, (SH)_2_) catalysts have been successfully synthesized by incorporating various functional groups in the ligands (Figure 5a). The ligand modification of UiO-66 was carried out to enhance the photocatalytic activity for the degradation of Rhodamine B under visible light. The results showed that UiO-66-NH_2_ and UiO-66-(OH)_2_ have narrow band gaps and good visible light absorption, but they cannot effectively catalyze the degradation of organic dyes, possibly due to their conduction band being close to the redox potential of Rhodamine B (Figure 5b) [106]. However, different results can be obtained when amino groups are incorporated in other MOFs. MIL-125-NH_2_ modified with amino functional groups can decompose 97% of Congo Red dye under 3 h of irradiation, while the original material MIL-125 can only degrade 60% pollute [107]. Three RE-PMOFs (BUT-224/225/226) with new topological structures were successfully obtained by introducing phenyl/pyridine groups in the middle of the porphyrin core (Figure 5c) and adjusting the symmetry and connectivity of the ligands. In addition, BUT-225 (Co) exhibits stronger CO_2_ absorption capacity and higher catalytic activity than other MOFs during CO_2_ reduction [108].

### 4.2. Construction of Donor-Acceptor System

The design of the internal structure of MOFs is an alternative strategy for high-powered photocatalysts. The donor-acceptor (D-A) system, which involves the interaction between electron-rich and electron-deficient organic components, has garnered significant interest from researchers. This system shows promising potential for optical and electrical materials. The unique electronic properties of the component endow the D-A system with different electron transfer behaviors between the donor and acceptor components [109,110], which lays the foundation for the charge transfer (CT) and energy transfer (ET) of MOFs containing the D-A system. The different connection or packaging of the donor and acceptor components will affect their mode of interaction, thereby determining the properties of the system.

**Figure 6:**
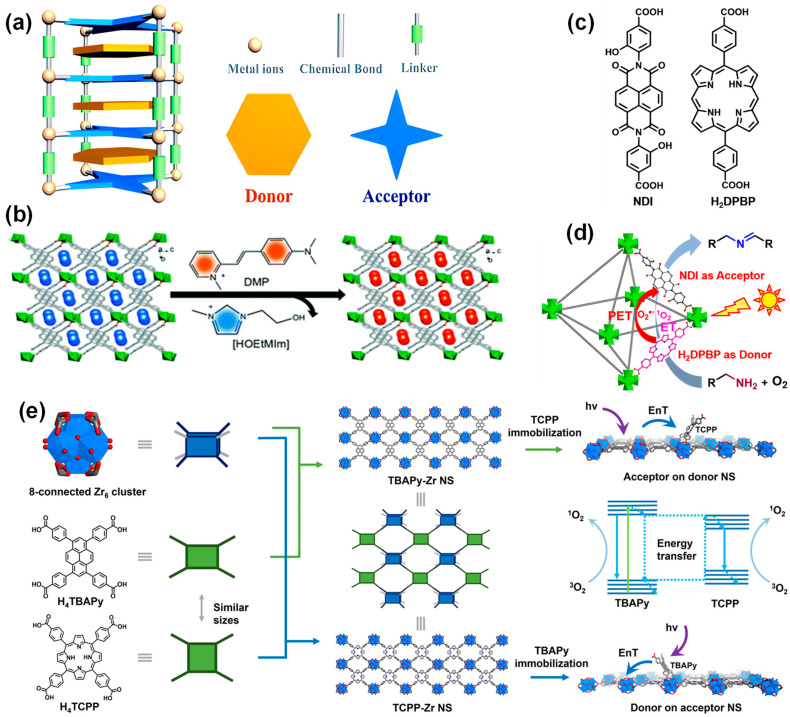
(**a**) Diagram of donor-acceptor-based MOF structure [112]; (**b**) schematic diagram of the ion exchange experiment by the incorporation of DMP cations where a, b and c represents the 3D spatial directions [114]; (**c**) chemical structure of the NDI and H_2_DPBP ligands [116]; (**d**) photocatalytic mechanism of amine coupling reaction over the mixed ligands Zr-NDI-H_2_DPBPMOF based on donor H_2_DPBP and acceptor NDI [116]; (**e**) schematic diagrams of the SBUs for the construction of TBAPy-Zr NS and TCPP-Zr NS (**left**), schematic illustration of the acceptor-on-donor-NS model (**right**, **top**), the EnT process (**right**, **middle**), and the donor-on-acceptor-NS model (**right**, **bottom**) [117].

Photo-induced electron transfer (PET) can be achieved by assembling electron donor and acceptor chromophores in a single MOF. Weak interactions, such as electrostatic and hydrogen bonding between the molecular entities of the donor and acceptor, provide a long-range ordered electron transfer channel and help to inhibit the recombination of charges [111]. In addition, MOFs provide an unprecedented level of controlling the D-A system layout through the design of metal nodes and organic linkers, the modularity of the framework topology, and the combination of different subjects (Figure 6a) [112]. The modularity and versatility of MOFs endow them with directional energy and charge transport by adjusting the geometric parameters, such as the distance or angle between the donor and acceptor (D-A), or by changing the electronic structure of the D-A system [113]. Additionally, the narrowed band gap of the material can be obtained by adjusting the highest occupied molecular orbital (HOMO) and the lowest unoccupied molecular orbital (LUMO) of the D-A system, thereby improving the performance of MOFs.

#### 4.2.1. Effect of D-A System in Promoting Light Harvesting

A donor-acceptor system containing MOFs with molecular-level heterojunctions was synthesized from a linear trinuclear Mn(II) cluster, a 1,1,3,6-tetraphenyl pyrene-based photoactive linker, and a D-π-A cationic dye cyanine (DMP) under isothermal conditions (Figure 6b) [114]. The interplay between the chromophore and energy acceptor, and the bandgap can be adjusted to a large extent. The resulting MOFs exhibited unique light absorption across the UV/visible to NIR light region, extremely high luminescence polarization anisotropy (0.97), and high light responsivity.

As a typical electron-rich chromophore molecule, porphyrin has a strong tendency to capture visible light. Extensive applications have been shown in homogeneous photocatalytic processes. Therefore, the introduction of porphyrins as building blocks into donor-acceptor system-containing MOFs shows promising prospects. Recently, several research teams have made significant advancements in developing D-A system-containing MOFs that are sensitive to visible light utilizing exceptional light-harvesting porphyrins. For example, the donor-acceptor system-containing MOFs constructed from H_2_TCPP and boron dipyrromethene can capture the light in the entire visible region, which greatly improves the utilization of solar energy. At the same time, efficient electron energy transfer from ligand to ligand was also observed between the pillared boron dipyrromethene (D) and porphyrin derivatives (A) [115].

#### 4.2.2. Effect of D-A System in Facilitating Electron Separation

The porphyrin-based MOF based on the donor-acceptor system also exhibits excellent performance in promoting electron separation. For example, a mixed ligand MOF (Zr-NDI-H_2_DPBP) photocatalyst was constructed using 5,15-bis (p-benzoic acid) porphyrin (H_2_DPBP) as the electron donor and naphthalene diimide (NDI) as the electron acceptor (Figure 6c). The prepared MOFs can produce high-efficiency photo-induced electron transfer, optimize charge separation efficiency, and thus improve photocatalytic performance (Figure 6d). The MOFs were used for the oxidation of different benzylamine derivatives in which the imine formation rate is 136 mmol g^−1^ h^−1^ higher than that of previously reported noble metal-free MOF photocatalysts [116].

Diverse ligands are used to construct the donor-acceptor system, and the catalytic performance of prepared MOFs can be affected by the functions and properties of the ligands. MOFs with high energy transfer efficiency can be constructed by selecting tetrakis (4-carboxyphenyl) porphyrin (H_4_TCPP) and 1,3,6,8-tetrakis(p-benzoic acid) pyrene (H_4_TBAPy) as the acceptor and donor parts, respectively. The results demonstrate that the energy transfer between the TBAPy donor and the TCPP acceptor can be fulfilled. The energy transfer efficiency of the TBAPy-Zr nanosheets with 22% TCPP reached 82% (Figure 6e). Compared to the unmodified MOFs, these MOF photocatalysts containing TCPP linkers exhibit a designed energy transfer process and reduced self-quenching as well as high photocatalytic activity by the introduction of an acceptor-on-donor system [117].

### 4.3. The Introduction of Co-Catalysts

**Figure 7:**
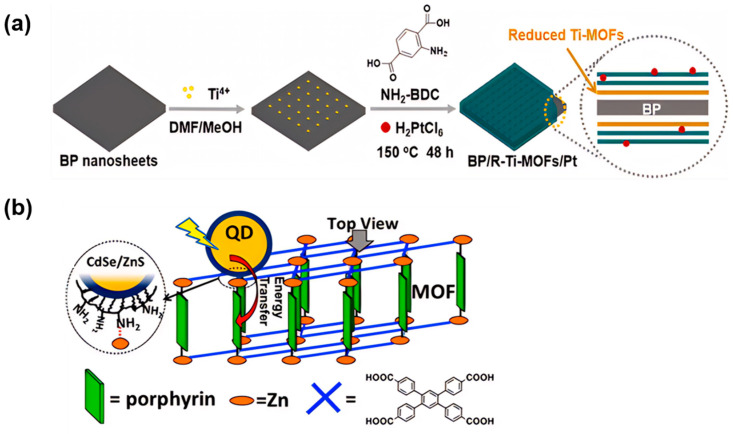
(**a**) Synthetic diagram of BP/R-Ti-MOFs/Pt hybrids [120]; (**b**) schematic diagram of a QD-MOF complex [121].

Photocatalysts are catalysts that can utilize light energy to catalyze reactions. However, the performance of photocatalysts may not be ideal in some applications, which makes it challenging to achieve efficient photocatalytic reactions. The introduction of co-catalysts is a recognized approach to enhance the performance of photocatalysts and amplify their advantageous effectiveness. Unlike optimizing the internal composition and structure design of MOFs, the introduction of co-catalysts can provide additional active sites to enhance the reaction rate. It can also modulate the band structure of the photocatalysts, alter their light absorption and photoelectron transfer properties, accelerate charge transfer, and suppress charge recombination [118,119]. For example, a hybrid MOF composed of platinum co-catalysts and black phosphorus (BP) was synthesized [120]. The prepared hybrids showed efficient charge transfer and excellent light absorption properties. The doping of platinum endows them with a narrowed band gap (Figure 7a), improves the harvesting efficiency of photo-induced electrons, inhibits the recombination of electrons and holes, and therefore improves the production efficiency of H_2_. The results show that the photocatalytic hydrogen production of titanium-based MOFs (BP/R-Ti-MOFs/Pt) with Pt co-catalysts is 10.3 times that of the original Ti-MOFs.

Besides, CdSe/ZnS core/shell quantum dots (QD) can also be used to functionalize porphyrin-based MOFs (Figure 7b). Energy transfer from QD to MOF can enhance the light collection ability of composite materials. QDs have a wide-range light absorption in the visible region, and the construction of QD-MOF hybrid structures can further expand their absorption in the solar spectral range. Time-resolved emission studies show that the energy is transferred from quantum dots to MOF after quantum dot excitation with efficiencies greater than 80%, suggesting effective enhancement of the light capturing and energy transfer ability for porphyrin-based MOFs by the introduction of the co-catalysts [121]. Qin and colleagues constructed GQDs@PCN-222 complexes by encapsulating graphene quantum dots into porphyrin-based metal-organic frameworks (PCN-222) for the reduction of CO_2_. The results showed that the activity of GQDs@PCN-222 was nearly four times that of pure PCN-222, and the rate of CO generation was up to 147.8 μmol g^−1^ h^−1^ under visible light irradiation [122].

## 5. Application of Porphyrin-Based MOFs in Photocatalysis

### 5.1. Photocatalytic Hydrogen Evolution

Hydrogen, a clean and efficient energy resource, produced from solar energy-driven water splitting exhibits great prospects [123,124,125,126]. The visible light absorption of MOFs is effectively broadened when porphyrin ligands are introduced. At the same time, specific active groups such as co-catalysts can be introduced into the larger pore size of MOFs, which endows porphyrin-based MOFs with great potential and advantages in using solar energy to produce hydrogen [127].

In 2012, Fateeva and coauthors [128] reported the photocatalytic hydrogen evolution over porphyrin-based MOFs. A water-stable porphyrin-based MOF (Al-PMOF) was constructed from the tetra (4-carboxy) porphyrin (H_2_TCPP) linker and the Al (OH)O_4_ chain node. The photocatalytic performance of Al-PMOF with free-base porphyrin and zinc metalloporphyrin in H_2_ evolution was studied. The MOF/EDTA/Pt system is adopted to harness the excited state energy of the porphyrin, namely, EDTA is used as the sacrificial electron donator and Pt as the co-catalyst to facilitate the reaction. The results indicated that Zn^2+^ can be introduced into the porphyrin core to form a zinc-porphyrin-based MOF. Both the porphyrin-based MOFs and the Zn-porphyrin-based MOFs can be used to produce hydrogen by a solar energy-driven water-splitting reaction.

The activity in photocatalytic hydrogen evolution of MOFs can be improved by the introduction and assembly of active metals in MOF frameworks. Porphyrin-based MOFs ([FeFe]@ZrPF) have high stability and high photocatalytic hydrogen production efficiency. In this system, the porphyrin unit is used as a photosensitizer, and the iron hydride enzyme is used as a hydrogen evolution catalytic site. The short distance between the two parts and the nature of chemical bonding allows for the fast transfer of photo-generated carriers. The chemical bonding within the materials can provide robust hydrogen elution systems by avoiding the use of electron mediators to transfer electrons from the photosensitizer to the catalyst [126].

**Figure 8:**
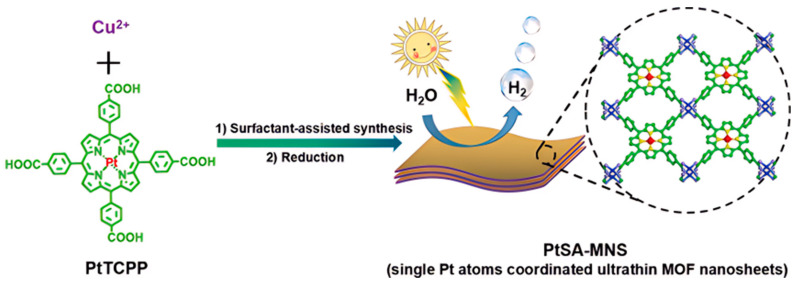
Synthesis of Pt single-atom-coordinated ultrathin MOF nanosheets (PtSA-MNSs) for photocatalytic hydrogen production [124].

The shape and structure of MOFs can also exert influence on their photocatalytic activity. Compared to bulk MOFs, MOFs with two-dimensional lamellar structures are more conducive to exerting the light absorption ability of porphyrin units in the framework, increasing the contact opportunity between the substrate and the active sites, and thus improving the catalytic activity. Two-dimensional MOF materials also show great potential in photocatalytic hydrogen evolution due to their ability to reduce the recombination of photo-generated electron-hole pairs. Ultrathin two-dimensional MOF nanosheets were constructed using metalloporphyrin (Pt@TCPP) as a linker and Cu_2_(COO)_4_ as a metal node through a surfactant-stabilized coordination strategy [124]. The preformed single Pt atom coordination porphyrin precursor is assembled into free-standing ultrathin metal-organic framework (MOF) nanosheets, in which a highly loaded single Pt atom is used for efficient photocatalytic H_2_ production (Figure 8). The single Pt atom-coordinated porphyrin-based MOFs achieved a new historical record in photocatalytic H_2_ production (11,320 μmol g^−1^ h^−1^) under visible light irradiation using ascorbic acid as the sacrificial agent and water as the solvent. The MOF nanosheets can be easily drop-casted onto a solid surface to create a thin film for significant practical application.

### 5.2. Photocatalytic CO_2_ Reduction

Fossil fuels including gasoline, oil, coal, and natural gas are used in large quantities every day, and excessive amounts of carbon dioxide are discharged into the air, which causes increasingly serious environmental problems [129,130,131,132]. Therefore, the collection and conversion of carbon dioxide into renewable energy fuels or high-value-added compounds have attracted great interest from scientists and entrepreneurs around the world, and various strategies for converting carbon dioxide into usable substances have been developed [133]. Among them, one of the most promising and economical methods is the photocatalytic reduction of carbon dioxide driven by solar energy [134,135]. For example, photocatalytic CO_2_ reduction can produce various fuels such as CO, formic acid, and hydrocarbons [103]. In recent years, MOFs have been widely studied for photocatalytic CO_2_ reduction due to their adjustable composition and structure, easy integration with other functional substances, and large surface area [136,137,138,139].

Synthesis of MOFs with appropriate organic linkers to promote light absorption is the first step in the photocatalytic reduction of CO_2_. So far, various organic linkers including porphyrin-based, metal complex-functionalized, and amine-functionalized organic linkers have been used to regulate the band gap of MOF photocatalysts. In 2013, Fu and coauthors [140] demonstrated for the first time that MOF photocatalysts can photocatalytically reduce CO_2_ to HCOO^−^. Researchers synthesized an aminated MOF photocatalyst using 2-aminoterephthalic acid as an organic linker and evaluated its photocatalytic performance in acetonitrile electrolyte with triethanolamine as the electron donator. The results confirm that the amino functionalization of MOFs promotes photocatalytic activity in CO_2_ reduction.

Porphyrin-based MOFs absorb visible light effectively and generate charge carriers, which make them suitable for photocatalytic CO_2_ reduction. For example, the porphyrin ligand in zirconium-based MOF PCN-222 serves as a visible-light-harvesting unit. It can suppress the detrimental, radiative electron-hole recombination owing to the presence of a deep electron trap state in PCN-222 and holes and improve the catalytic activity of photo-reduced CO_2_ [141].

The stability of MOFs can be enhanced by in situ substitution of porphyrin. A labile MOF (BUT-109(Zr)) was converted to a stable porphyrin-based MOF (BUT-110) by replacing a naphthalene diimide dibenzoate (NDIDB^2−^) with a size- and geometry-matching porphyrin ligand (DCPP^2−^). Compared with BUT-109(Zr), BUT-110 MOFs with different porphyrin contents showed various chemical stability enhancements. The obtained BUT-110 material exhibited excellent stability at a wide pH range (1–10) when the porphyrin content exceeded 50%. Moreover, the BUT-110 series with metalloporphyrin ligands showed better photocatalytic activity in the CO_2_ reduction to CO. By adjusting the type and content of metal porphyrins in BUT-110, BUT-110-50%-Co is believed to be the best compromise in terms of chemical stability, photocatalytic efficiency, and synthesis cost [101].

The MOFs with two-dimensional lamellar structures also show good catalytic ability in CO_2_ reduction. An ultra-thin two-dimensional zinc-porphyrin-based metal-organic framework (Zn-MOF nanosheets) photocatalyst exhibited excellent photocatalytic activity and high CO selectivity under mild conditions because of its better charge transfer ability and longer lifetime of photo-generated charges. The conversion number (TON) of CO is 26.2 within 6 h in the presence of Zn-MOF nanosheets, indicating their stronger CO_2_ reduction ability [142].

**Figure 9:**
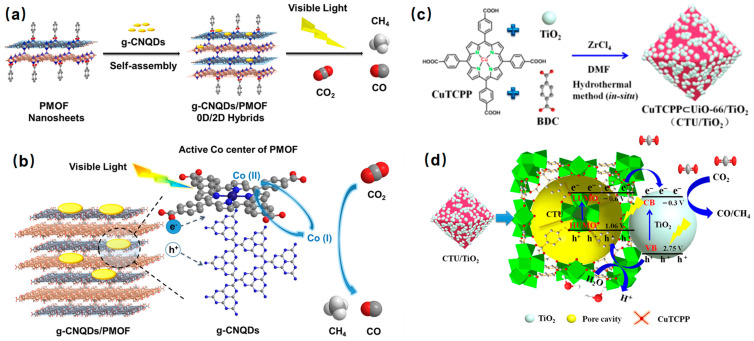
(**a**) Preparation of g-CNQDs PMOF hybrids [143]; (**b**) carbon dioxide reduction diagram over g-CNQDs PMOF hybrids [143]; (**c**) schematic diagram of CTU/TiO_2_ synthesis route [144]; (**d**) pathway of photocatalytic CO_2_ reduction over CTU/TiO_2_ hybrids [144].

To further improve their ability of photocatalytic CO_2_ reduction, porphyrin-based MOFs can also be integrated with other functional materials to construct photocatalytic composites. As shown in Figure 9a, a new hybrid catalyst was formed from the combination of two-dimensional (2D) ultrathin porphyrin MOFs and zero-dimensional (0D) carbon nitride quantum dots (g-CNQDs). g-CNQDs are coordinated with Co active sites in PMOF, which greatly shortens the migration pathways of photo-generated charges and gaseous substrates (Figure 9b). Effective separation of electron-hole pairs and long-life capture of electrons in the Co center endow the material with enhanced activity in photocatalytic CO_2_ reduction and improved selectivity to CH_4_. The average lifetime (τ) of g-CNQDs/PMOF with a feed ratio of 1:6 is 5.25 ns, much lower than 7.73 ns of g-CNQDs. This indicates the highest electron transfer efficiency from g-CNQDs to PMOF and the highest inhibiting degree of the recombination of photo-generated carriers. The effective separation of electron-hole pairs generated by this material and the long-lived captured electrons in the Co center result in a 2.34-fold improvement in the CO generation rate (16.10 μmol g^−1^ h^−1^) compared to the pristine PMOF. The methane (CH_4_) generation rate increased 6.02 times (6.86 μmol g^−1^ h^−1^) and at the same time, the selectivity of CH_4_ was also enhanced [143]. In addition, CuTCPP-UiO-66/TiO_2_ (CTU/TiO_2_) composites (Figure 9c) were prepared by immobilizing Cu(II) (4-carboxyphenyl) porphyrin into an UiO-66 MOF structure through a coordination mode. The composite exhibited unusual activity in CO_2_ reduction in which the released CO was about seven times that of pure TiO_2_. The light-induced carriers in the composites were separated between the MOF and TiO_2_ components, which promoted the separation of electron-hole pairs (Figure 9d). Therefore, CTU/TiO_2_ showed good catalytic activity and recyclability in the process of photocatalytic reduction of CO_2_ to CO [144].

### 5.3. Photocatalytic Synthesis of Organic Compounds

Singlet oxygen is a reactive oxygen species that can be used for many catalytic transformations [145,146,147,148,149]. For the photocatalysts, their ability to effectively generate singlet oxygen and prolong its lifetime is crucial for their use in photooxidation reactions such as sulfide oxidation and amine oxidation.

**Figure 10:**
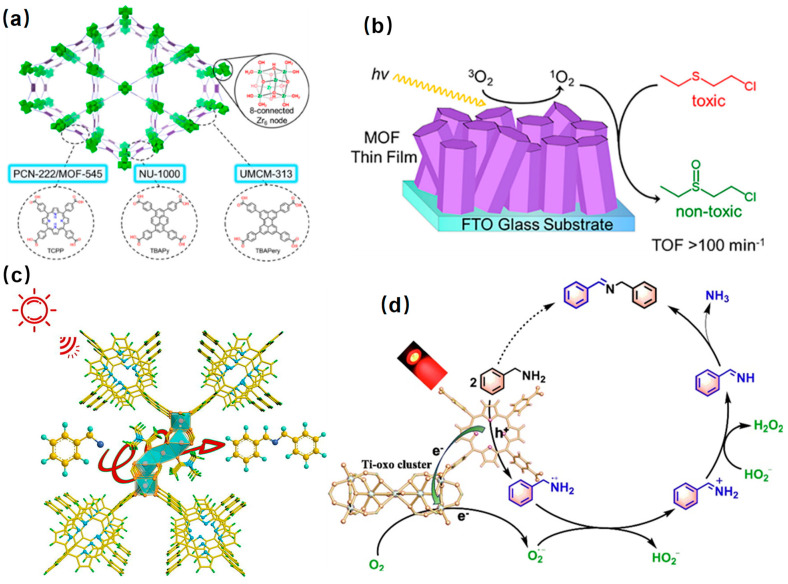
(**a**) Structure of NU-1000, PCN-222/MOF-545, and UMCM-313 and their corresponding linkers and common Zr_6_-node [148]. (**b**) Mechanistic diagram of photocatalytic oxidation of 2-chloroethyl ethyl sulfide over the MOF thin film [148]; (**c**) selective photocatalytic oxidation of amines on Ti-PMOF-DMA under red LED irradiation [154]; (**d**) mechanism of selective photocatalytic aerobic oxidation of benzylamine over Ti-PMOF-DMA [154].

Sulfoxide is an indispensable intermediate in pharmaceutical, agrochemical, and other fine chemical industries [150,151]. The thionyl group in the sulfoxide molecule is chemically unstable and easily overoxidized to sulfone. Strong oxidants such as potassium permanganate or dichromate are usually used for the oxidation of sulfoxide, resulting in serious environmental pollution [152]. Porphyrin-based metal-organic frameworks have remarkable advantages in the photocatalytic oxidation of sulfoxide due to their wide absorption from ultraviolet to the visible light region, high chemical stability, and environmental friendliness. PCN-222/MOF-545, NU-1000, and UMCM-313, MOFs with porphyrin, pyrene, and perylene ligands, respectively, were used as photocatalysts to complete the oxidation of 2-chloroethyl ethyl sulfide (CEES) using the generated singlet oxygen under LED irradiation (Figure 10a,b) [148]. The results show that the oxidation rate of CEES is positively correlated with the singlet oxygen quantum yield which is dependent on the organic linker of Zr-based MOF, that is, porphyrin (PCN-222/MOF-545) < pyrene (NU-1000) < perylene (UMCM-313). Additionally, the strong coordination between metal atoms and porphyrins improves their chemical stability and simplifies the recovery process. Porphyrin-based MOFs (AgTPyP) with two-dimensional structures produce singlet oxygen effectively, and the conversion and selectivity are close to 100%. Moreover, the selectivity and conversion efficiency of AgTPyP were not reduced after five cycles, indicating their excellent stability and reusability [153].

Imines are important biomedical intermediates with excellent pharmacological and biological activities. The Ti-porphyrin-based metal-organic framework (Ti-PMOF-DMA) was synthesized by solvent-controlled synthesis, showed excellent catalytic activity, and was selective in the photocatalytic aerobic oxidation of benzylamine. The results showed that benzylamine could be oxidized to N-benzylbenzaldehyde diamine in 40 min, achieving 94% conversion and 88% selectivity (Figure 10c,d). At the same time, the porphyrin-based MOF also showed high stability [154].

It is reported that the photocatalytic ability of porphyrin-based MOFs can be improved by prolonging the lifetime of singlet oxygen. For example, the presence of certain metal dots can promote the conversion of the energy of the excited photocatalyst to ground-state oxygen and enhance the spin-orbit coupling effect, thereby generating singlet oxygen. The lifetime of singlet oxygen produced by MOF-Eu-TPPS composed of TPPS bridged rare earth material is 23 μs, which is longer than that of TPPS (4 μs) inserted in Eu-based layered double hydroxides (LDH) in oxygen. It is confirmed that the coordination and rigid separation in MOF will enhance the production of singlet oxygen. The extension of singlet oxygen lifetime over porphyrin-based MOFs provides a more efficient approach to photocatalytic synthesis and conversion [155].

### 5.4. Photocatalytic Removal of Pollutants

Environmental issues related to clean energy and pollution have become increasingly prominent. In the past few decades, a large quantity of pollutants has been discharged into the environment due to urbanization and population growth [156,157,158,159]. Organic dyes pose a serious threat to the environment and are difficult to remove from the environment due to their varying resistance to photodecomposition and oxidation [160]. So far, many technologies have been developed and used to remove harmful pollutants in the environment, such as adsorption [161], biological oxidation [162], chemical treatment, and photo-degradation [163,164]. Among them, photocatalytic methods have shown high selectivity for organic pollutants, minimal damage to ecosystems, and are usually low cost [165,166,167]. Particularly, porphyrin-based MOFs have a wide light response range and easily regulated structure and function, which endow them with clear advantages in treating pollutants by the utilization of solar energy.

MOF-525 and MOF-545 comprised of Zr-oxide clusters and porphyrin moieties in different geometries can be applied to the adsorption and removal of an organic pollutant sulfamethoxazole (SMX). Both MOFs showed adsorption ability to SMX due to their mesoporous structure. The adsorption kinetic rate of MOF-545 with mesopores was about 20 times that of MOF-525 micropores, although their maximum adsorption capacities are 690 and 585 mg/g for MOF-545 and MOF-525, respectively. The π-π interactions and hydrogen bonding between the SMX molecule and the N sites of the porphyrin units control the adsorption of SMX on the porphyrin-based MOFs. The adsorbent is easy to regenerate after washing with acetone and can be reused for four adsorption-desorption cycles without a significant decrease in performance [168].

Pollutant removal can be improved by strengthening the adsorption of organic pollutants on the materials by incorporating other materials with porphyrin-based MOFs. For example, Zr-porphyrin-based MOFs (PCN-224-TiO_2_) were synthesized by porphyrin, ZrCl_4_, and nano-TiO_2_. They showed significant visible light absorption and high photocurrent. Compared with the mixture of TiO_2_ (TCPP/TiO_2_) and PCN-224, PCN-224-TiO_2_ exhibited higher performance in the removal of methylene blue under visible light. The removal rate of methylene blue reached 93.2% within 4 h of low-power light irradiation. Moreover, the PCN-224-TiO_2_ photocatalyst was kept stable during the cycling reactions [169].

**Figure 11:**
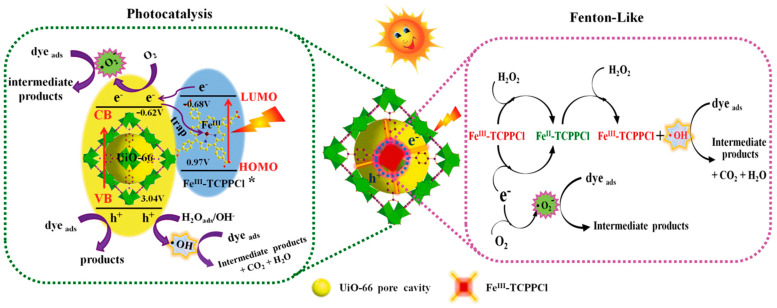
The proposed photocatalytic mechanism of Fe^III^-TCPPCl⊂UiO-66 co-catalytic Fenton-like reaction [171].

In addition, the photocatalytic degradation efficiency of organic pollutants over porphyrin-based MOFs can be regulated by the selection and assembly of porphyrin ligands and metal nodes. PCN-224 (Zr/Ti) with bimetallic nodes was synthesized by a simple cation exchange method to partially replace Zr with Ti in the Zr-porphyrin-based MOF (PCN-224). The partial replacement with Ti in the MOFs allows faster electron transfer from TCPP to oxygen clusters, enhanced photocurrent, and significantly improved degradation efficiency of pollutants [170]. Fe^(III)^-TCPPCl⸦UiO-66 MOF, a derivative of the zirconium-based metal-organic framework, achieved a 100% degradation of organic pollutant rhodamine B [171]. The mixed ligand Fe^III^-TCPPCl that was incorporated in the pore of UiO-66 not only acted as a photosensitizer that promoted light absorption and transfered electrons from the excitic state of Fe^III^-TCPPCl (Fe^III^-TCPPCl*) to the conductive band of UiO-66 rapidly to suppress the recombination of photo-induced charges over the combined structure, but also functioned as a co-catalyst to decompose hydrogen peroxide to hydroxyl radicals (Figure 11). In addition, porphyrin-based MOFs also showed excellent removal effects on inorganic pollutants. The Zr-MOF-SH/MF composites can be used to selectively remove Hg^2+^ from water with an adsorption capacity of 412.5 mg g^−1^. Moreover, the Zr-MOF-SH/MF composites could be used to simultaneously remove oil and Hg^2+^ from water, and the removal efficiency of Hg^2+^ increased with the enhancement of oil content in the oil-water mixture. In addition, the composites can be recycled and reused five times, in which only a slight decrease in adsorption was observed [172].

### 5.5. Photocatalytic Nitrogen Fixation

Nitrogen fixation, as it can convert atmospheric nitrogen into nitrogen-containing molecules that can be used by all living organisms including humans, has been regarded as one of the most important natural processes in the Earth’s nitrogen cycle. For example, ammonia (NH_3_) is the most common industrial chemical and carbon-free energy storage molecule [173]. However, the industrial preparation of NH_3_ is a highly energy-intensive and environmentally unfriendly process. Therefore, an alternative sustainable method for efficient generation of NH_3_ from N_2_ under mild conditions is sorely needed to alleviate the increasing energy and environmental issues. Porphyrin-based MOFs are highly porous crystalline materials with large surface areas and highly ordered pores, which can effectively enhance N_2_ adsorption. Porphyrin molecules, as a strong photosensitive linker, can significantly broaden the light response of MOFs. Furthermore, the photocatalytic nitrogen fixation efficiency can be significantly improved by rationally designed porphyrin-based MOFs to enhance the separation of photo-generated charges [68]. Therefore, porphyrin-based MOFs exhibit excellent performance and a positive future in photocatalytic nitrogen fixation.

**Figure 12:**
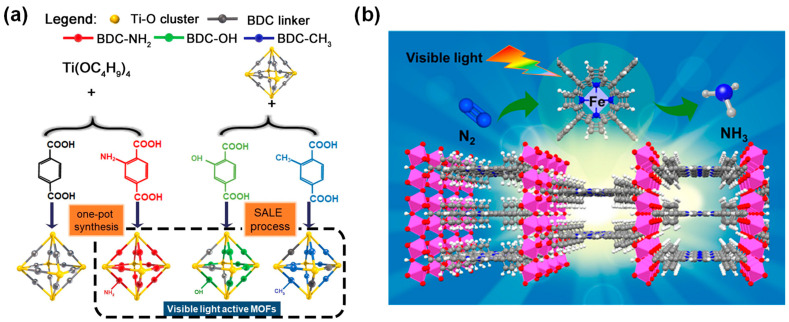
(**a**) Schematic diagram of the targeted visible light active MOFs for photocatalytic N_2_ fixation [174]. (**b**) The structure of Al-PMOF(Fe) [175].

Until now, few investigations about the utilization of MOFs for photocatalytic nitrogen fixation have been reported. For example, a comparative study of Ti-based isostructural MOFs (NH_2_-MIL-125 (Ti), OH-MIL-125 (Ti), and CH_3_-MIL-125 (Ti)) showed that NH_2_-MIL-125 (Ti) (Figure 12a) had the optimal performance in nitrogen reduction where the amine-functionalized linker effectively enhanced the light absorption and the Ti^3+^ sites increased the efficiency of N_2_ adsorption and reduction [174]. Porphyrin molecules have strong light absorption capabilities and can be used as linkers to construct MOFs, endowing them with excellent performance in photocatalytic nitrogen fixation. For example, Shang et al. developed a porphyrin-based metal-organic framework (PMOF) with Fe as the active center, serving as an artificial photocatalyst for the nitrogen reduction reaction at room temperature (Figure 12b). Porphyrin is an efficient light-harvesting ligand, Al serves as the metal node to stabilize the MOF frameworks, and Fe acts as the active center for N_2_ adsorption and reduction. The Al-PMOF (Fe) exhibits excellent performance in visible-light-driven nitrogen fixation under a N_2_ flow rate of 20 mL min^−1^ and reaches an ammonia production rate of 127 μg g^−1^ h^−1^, which is 50% higher than that of Al-PMOF. The reason for this is the insertion of Fe into the porphyrin ring which endows Al-PMOF(Fe) with a higher charge separation efficiency and faster interface charge transfer speed. In addition, electrons will quickly transfer from the free porphyrin ring to the inserted Fe, which effectively suppresses the recombination of charge carriers [175].

## 6. Conclusions and Foresight Perspective Outlooks

In the context of the gradual depletion of global fossil energy and increasingly serious environmental pollution, it is of great significance to develop high-powered photocatalysts to efficiently utilize solar energy and remove pollutants. Porphyrin-based MOFs, integrating versatile functional porphyrins and porous and adjustable MOFs in which metal or metal-oxide clusters through carboxylate or nitrogen-containing heterocyclic linkers, are believed to be an ideal platform for photocatalysis due to their precisely tailorable structures and properties. Moreover, their photocatalytic performance can be further improved by the modification of porphyrin ligands, construction of donator-acceptor systems, and introduction of co-catalysts. Thus, the well-designed porphyrin-based MOFs show excellent photocatalytic performance and great potential in the fields of hydrogen evolution, organic synthesis, CO_2_ reduction, and pollutant removal.

However, some challenges limit the development of porphyrin-based MOFs. The first challenge is the high cost and low yield of porphyrin-based MOFs, which are well aligned with those in MOFs in general. Especially for asymmetric porphyrin ligands, their production usually requires a complicated synthesis process and laborious purification in addition to expensive raw materials, which hinders their wider application. Although immobilizing atomic-scale co-catalysts in MOFs can reduce the use of expensive metals, further efforts are required to decrease the cost of materials. The second challenge is their limited structural stability, especially in some harsh conditions such as high temperatures, strong light, strong acids or alkalis, and so on. The third challenge is their relatively poor conductivity. It is necessary to study the charge carriers, electron mobility, and conductivity mechanism [176]. The mechanism of charge transfer of porphyrin-based MOFs with different composites (or structures) in particular needs special attention and more effort. Finally, high-symmetry orientation design is crucial for the design and construction of porphyrin blocks, which prevents the expansion of porphyrin-based metal-organic frameworks and affects their potential catalytic applications. Therefore, exploring the structure-activity relationship of porphyrin-based metal-organic framework materials is of great significance for the design and synthesis of such materials. In general, porphyrin-based MOFs with a low cost, structure robustness, high conductivity, and low limitation on structure symmetry will have a promising future [177].
